# Simple, Rapid and Reliable Preparation of [^11^C]-(+)-α-DTBZ of High Quality for Routine Applications

**DOI:** 10.3390/molecules17066697

**Published:** 2012-06-01

**Authors:** Jinming Zhang, Xiaojun Zhang, Yungang Li, Jiahe Tian

**Affiliations:** Department of Nuclear Medicine, the PLA General Hospital, Beijing 100853, China

**Keywords:** [^11^C]-(+)-α-DTBZ, VMAT2, position emission tomography, automated synthesis

## Abstract

[^11^C]-(+)-α-DTBZ has been used as a marker of dopaminergic terminal densities in human striatum and expressed in islet beta cells in the pancreas. We aimed to establish a fully automated and simple procedure for the synthesis of [^11^C]-(+)-α-DTBZ for routine applications. [^11^C]-(+)-α-DTBZ was synthesized from a 9-hydroxy precursor in acetone and potassium hydroxide with [^11^C]-methyl triflate and was purified by solid phase extraction using a Vac tC-18 cartridge. Radiochemical yields based on [^11^C]-methyl triflate (corrected for decay) were 82.3% ± 3.6%, with a specific radioactivity of 60 GBq/μmol. Time elapsed was less than 20 min from end of bombardment to release of the product for quality control.

## 1. Introduction

The vesicular monoamine transporter (VMAT) has two pharmacologically distinct isoforms, VMAT1 and VMAT2 [1]. In contrast to VMAT1, VMAT2 is primarily found in the central nervous system of rodents and humans. Over 95% of striatal VMAT2 binding sites are associated with dopaminergic terminals, and the striatal VMAT2 binding site density is a linear function of the mesencephalic nigrostriatal neuron number [2]. Type-2 vesicular monoamine transporter binding is predicted to be unaffected by dopaminergic agents or synaptic dopamine levels because synaptic vesicle function is apparently regulated by transfer of vesicles between the reserve and actively cycling synaptic terminal pool with relatively stable vesicle numbers [2]. Experimental evidence is consistent with this prediction; VMAT2 binding is not affected by dopaminergic drug treatments that cause changes in dopamine receptor or DAT expression [3,4]. VMAT2 has been used to monitor and diagnosis neurodegenerative disorders such as Parkinson’s and Huntington’s disease [5]. More recently, VMAT2 has been found expressed in human islet beta cells in the pancreas, and its use as a surrogate marker for beta cell mass loss and progression of diabetes has been suggested [6]. Thus, imaging VMAT2, particularly with high affinity PET, is an area of ongoing research interest.

(+)-α-Dihydrotetrabenazine [(+)-α-DTBZ] is the ideal ligand for VMAT due to its high affinity for VMAT2 and lipophilicity, confirmed by pharmacological results of *in vitro* studies. In the early 1990s, [^11^C]-(+)-α-DTBZ was investigated as a tracer for imaging VMAT2. Lang and Tong *et al*. demonstrated the correlation between the DTBZ to VMAT2 binding levels in the stratum [7,8].

[^11^C]-(+)-α-DTBZ was superior to other tracers, such as [^18^F]-DOPA or [^18^F]-CFT in studies of dopaminergic teriminals. The number of VMAT2 vesicles is not affected by pharmacological treatment with dopaminergic analogues which affect other elements of the system, such as the dopamine, DAT and D2 receptor. The simple synthesis of [^11^C]-(+)-α-DTBZ was developed by Jewett, who reacted a 9-hydroxy precursor with [^11^C]-methyl iodide on a column of alumina impregnated with KOH [9]. The KOH-impregnated alumina column was dried of atmospheric moisture to avoid failure of the synthesis. Quincoces later described the use of an alumina solid phase extraction cartridge to replace the KOH-alumina column, which was eluted with ethyl ether and ethanol [10]. The residual solvents were removed by evaporation. This method was not used due to low radiochemical purity. Instead, we attempted to use a C-18 SPE cartridge rather than an alumina SPE cartridge to enable reliable, high quality preparations of [^11^C]-(+)-α-DTBZ for routine applications.

## 2. Results and Discussion

### 2.1. Production

[^11^C]-(+)-α-DTBZ synthesis was automated using a cartridge purification method. Briefly (+)-desmethyldihydrotetrabenazine and 3 M KOH in DMSO was mixed with [^11^C]-methyl triflate as a methylation agent. After reaction and purification with Sep-Pak Vac tC-18 and an alumina cartridge, the radiochemical yield of [^11^C]-(+)-α-DTBZ was 82.3 ± 3.6% based on [^11^C]-methyl triflate (corrected for decay). It took about 20 min from the end of bombardment to release of the product for quality control. About 5.5 ± 0.4 GBq of [^11^C]-(+)-α-DTBZ can be obtained after a bombardment time of 10 min.

[^11^C]-methyl iodide was also used as the methylating agent for [^11^C]-(+)-α-DTBZ to obtain the same synthesis yields as those observed for [^11^C]-methyl triflate. However, [^11^C]-methyl iodide produced products of inferior radiochemical purity when compared with those prepared using [^11^C]-methyl triflate. Because [^11^C]-methyl triflate is more reactive than [^11^C]-methyl iodide, it is washed more easily from Sep-Pak, ultimately leading to a product of superior radiochemical purity.

### 2.2. Purification with Sep-Pak C-18

The reaction solution was passed through different Sep-Pak C-18 cartridges after mixing with 10 mL of water. The Sep-Pak C-18 was washed with 10 mL water. The Sep-Pak cartridges used were Waters C-18, tC-18 and Vac tC-18, respectively. The product was eluted from Sep-Pak C-18 with 1 mL ethanol. The elution yields were calculated from radioactivity remaining on the Sep-Pak and the radioactivity measured in the product fraction. The break-out efficiency was determined by analyzing the wash fractions (waste) by HPLC for “break-out” from the SPE cartridges. The results are shown in Table 1. 

**Table 1:** Elution and break-out efficiencies with different Sep-Pak C-18.

Sep-Pak	Elution yields (%)	Break out (%)
C-18	22.1 ± 7.8	0.5 ± 0.4
tC-18	55.6 ± 10.2	1.2 ± 0.6
Vac tC-18	95.3 ± 2.6	2.5 ± 1.4

**Figure 1:**
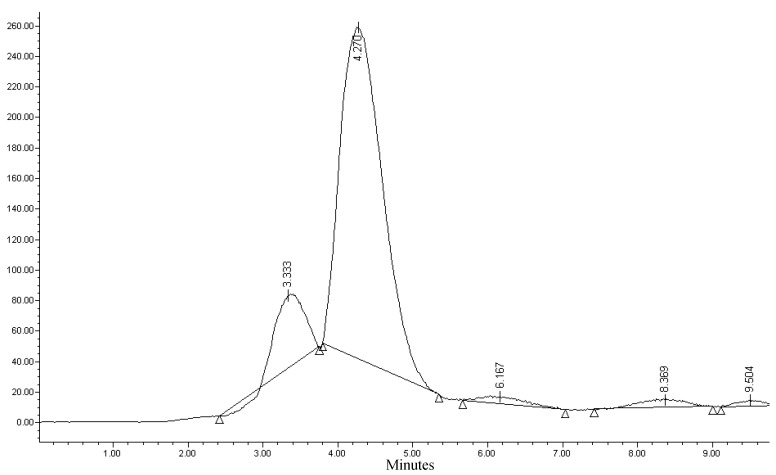
RCP of [^11^C]-(+)-α-DTBZ by two alumina N Sep-Pak purification.

The Sep-Pak C-18 and tC-18 remained highly radioactive, which indicated low elution yields. We selected the Sep-Pak Vac tC-18 as the best purification column, as it had a high elution yield and low break-out. The final preparation was not clear but yellow. By placing an alumina Sep-Pak cartridge immediately before the filter, we were able to eliminate the yellow colored impurities. The final preparation was clear. Quincoces [10] used two Sep-Pak alumina N cartridges as purification columns, and 5 mL of ethyl ether with 1% ethanol were eluted in two steps from the column. We were unable to reproduce these results, as the observed radiochemical purity was less than 75% (Figure 1).

### 2.3. Quality Control

**Figure 2:**
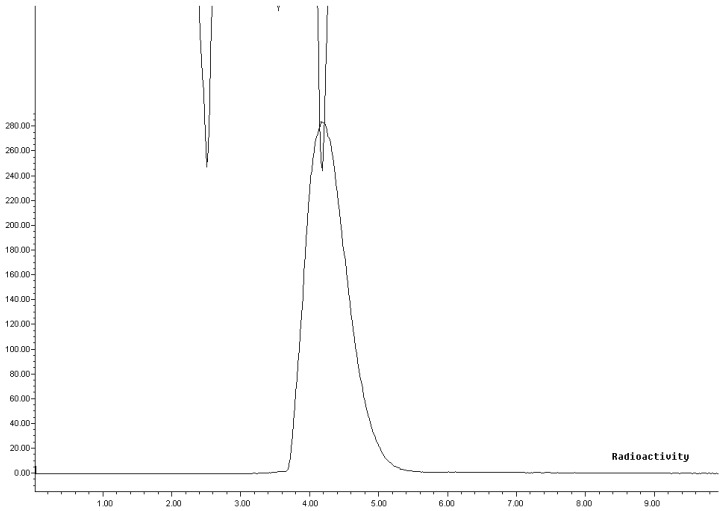
RCP of [^11^C]-(+)-α-DTBZ analysised by HPLC (UV was standard).

The radiochemical purity was determined by HPLC; the desired product eluted as a single peak with a retention time of 4.2 min, which corresponded exactly with the observed UV retention of cold DTBZ standard at 254 nm. (Figure 2).The radiochemical purity was >99%. The specific radioactivity was 60 GBq/μmoL (n = 12).

### 2.4. Biodistribution of [^11^C]-(+)-α-DTBZ in Normal Mice

NH mice were sacrificed at 0, 10, 20, 30, 60 min post-injection of [^11^C]-(+)-α-DTBZ. Radioactivity passed through the blood-brain barrier [BBB] as indicated by cortex uptake of 12.1% ± 1.86% ID/g, and the striatum of 19.18% ± 1.25% ID/g immediately post-injection. As expected, the ratio of striatum to cortex increased to about 3.9 at 20 min and remained 3.4 at 30 min. The radioactivity distribution (Table 2) was greatest at the liver with excretion occurring through the kidney.

**Table 2:** Biodistribution of [^11^C]-(+)-α-DTBZ in mice (mean ± SD, n = 5, %ID/g).

	0 min	10 min	20 min	30 min	60 min
Blood	12.10 ± 1.85	1.83 ± 0.19	1.93 ± 0.30	1.13 ± 0.07	1.33 ± 0.06
Heart	20.87 ± 2.40	3.81 ± 0.69	2.56 ± 0.66	1.82 ± 0.12	1.43 ± 0.08
Liver	2.85 ± 0.20	10.27 ± 1.47	13.16 ± 2.26	9.50 ± 0.99	10.61 ± 1.69
Spleen	1.79 ± 0.51	5.36 ± 0.59	5.35 ± 0.69	3.19 ± 0.15	2.57 ± 0.28
Lung	27.24 ± 9.56	5.03 ± 0.63	5.70 ± 0.30	3.34 ± 0.33	1.85 ± 0.44
Kidney	3.32 ± 1.30	7.57 ± 0.87	7.62 ± 1.12	4.70 ± 0.04	3.42 ± 0.19
Striatum	19.17 ± 1.25	13.00 ± 0.29	10.85 ± 1.56	5.75 ± 0.39	2.32 ± 0.16
Cortex	16.29 ± 1.97	3.42 ± 0.08	2.76 ± 0.44	1.71 ± 0.10	0.93 ± 0.11
Striatum/cortex	1.2	3.8	3.9	3.4	2.5

## 3. Experimental

### 3.1. Materials

1,3,4,6,7,11β-Hexahydro-10-methoxy-3-(2-methylpropyl)-(2*R*,3*R*,11b*R*)-2*H*-benzo[a]quinolizine-2,9-diol), the precursor for 2*H*-benzo[a]-quinolizine-2-ol,1,3,4,6,7,11b-hexahydro-9,10-dimethoxy-3-(2- methylpropyl)-(2*R*,3*R*,11b*R*)-[^11^C]-(+)-α-dihydroterabenazine, the reference standard for [^11^C]-di- hydroterabenazine; and 1 M lithium aluminum hydride in THF were purchased from ABX Compounds (Radeberg, Germany). 57% HI, DMSO and acetone were obtained from Aldrich (city, country) and used without further purification. Sep-Pak C-18 (400 mg), tC-18 (400 mg), Vac tC-18 (500 mg) and Alumina cartridges (1 g) were purchased from Waters (Milford, MA, USA), 0.22 μm sterile filters were purchased from Millipore (Billerica, MA, USA). The analytical HPLC column (C-18, 250 × 4.6 mm) was obtained from Phenomenex (Torrance, CA, USA).

### 3.2. Synthesis of ^11^C-(+)-α-DTBZ

**Scheme 1:**
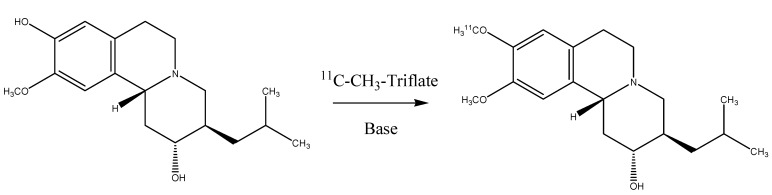
Synthesis route of ^11^C-(+)-α-DTBZ.

This was accomplished as shown in Scheme 1. (+)Desmethyldihydrotetrabenazine (0.8 mg) in DMSO (0.2 mL) with 3 M KOH (4 μL) was mixed with [^11^C]-methyl triflate as a methylation agent. Purification was accomplioshed using a Sep-Pak Vac tC-18 and an alumina cartridge.

### 3.3. Fully Automated Radiosynthesis

**Figure 4:**
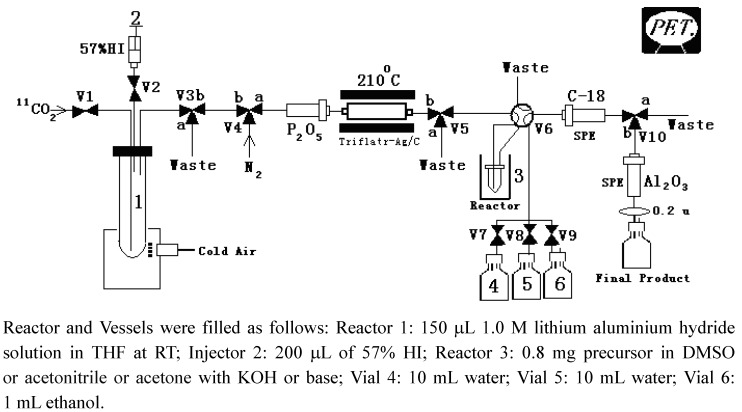
A scheme of the synthesis module.

A scheme of the synthesis module is presented in Figure 4. All details given in the following section refer to this Figure.

Three kinds of C-18 cartridge were preconditioned using ethanol and water; one of them was connected between the V6 and V10 position. The alumina cartridge was treated with water and connected between V10 and the collection vial.

### 3.4. Instruments

For the fully automated preparation of [^11^C]-DTBZ, a Methiodine Module and Methylation Module (PET Beijing Science and Technology Co., Ltd., Beijing, China) were remotely controlled by a standard PC. Analytical HPLC was performed on the following systems: Waters 515 pump, 2487 UV-detector, and BioScan Flow-Count (Washington, DC, USA). 

### 3.5. Production of [^11^C]-Methyl Iodide and [^11^C]-Methyl-Triflate [11,12]

Irradiation of a 99.5/0.5 mixture of N_2_/O_2_ (v/v) with a 20 Mev proton beam at 40 μA in a Sumitomo HM-20S (Osaka, Japan) cyclotron for 10 min yielded about 30 GBq of [^11^C]-carbon dioxide. At the end of bombardment, the [^11^C]-CO2 was passed through stainless steel tubes to the synthesis unit. The [^11^C]-CO2 was frozen in a stainless steel loop at −190 °C using liquid nitrogen. The frozen [^11^C]-CO2 was warmed up to −20 °C and transferred by a stream of nitrogen gas at 15 mL/min to the reactor containing 150 μL 1.0 M lithium aluminium hydride solution in THF at RT. The reactor was heated to 170 °C to remove the THF by a stream of nitrogen gas at 40 mL/min. After cooling the reactor, 200 μL of 57% HI was added and the [^11^C]-methyl iodide was carried by a stream of nitrogen gas. The [^11^C]-methyl iodide was passed over a silver triflate/C on-line with the gas/solid exchange reaction at 210 °C yielding the [^11^C]-methyl triflate. 

### 3.6. Automated On-Line Synthesis of [^11^C]-(+)-α-DTBZ

Gaseous [^11^C]-methyl triflate produced by the Methiodine Module was delivered through V6 into the reactor of the previously prepared synthesizer system and trapped on-line in the reaction mixture containing (+)-desmethyldihydrotetrabenazine (0.2 mg) in DMSO or acetone (0.2 mL) with KOH or NaOH. The methylation reaction was completed in three minutes according to the reaction illustrated in Figure 4. After the reaction, the solution was passed through Sep-Pak C-18 after mixing with 10 mL water in Vial 4. The Sep-Pak C-18 was then washed with 10 mL water in Vial 5. The preparation was eluted from Sep-Pak C-18 with 1 mL ethanol, passed through an alumina cartridges, reconstituted with 9 mL saline and filtered through 0.22 μm filter.

### 3.7. Quality Control

Chemical and radiochemical impurities were detected using radio-HPLC. Quality was assessed with analytical RP-HPLC; acetonitrile and 0.1 M ammonium formate (25/75; v/v, pH = 4.0) were used as the mobile phase with a flow rate of 1 mL/min. The entire quality control was completed within 8 min; the retention time of the precursor (Nor-DTBZ) was 3.5 min and the product [^11^C]-(+)-α-DTBZ was eluted with a retention time of 4.2 min. The chemical identity of [^11^C]-(+)-α-DTBZ was determined by co-injection of the unlabelled reference compound, (+)-α-DTBZ.

### 3.8. Biodistribution of [^11^C]-(+)-α-DTBZ in Normal Mice

[^11^C]-(+)-α-DTBZ (0.2 mL, 3.7 MBq) was injected through the tail vein into five groups of NH mice (20~22 g, n = 5/group). The mice were sacrificed at 0, 10, 20, 30, and 60 min post-injection. The organs (blood, heart, liver, spleen, lung, kidneys, striatum and cortex) were dissected and weighed, prepared for counting with a well-type Na(I) detector, and the uptake of each organ was expressed as the percentage of injection dose per gram (%ID/g). All biodistributions were carried out in compliance with the national laws related to the conduct of animal experimentation.

## 4. Conclusions

An automated [^11^C]-(+)-α-DTBZ synthesis based on a Sep-Pak Vac tC-18 cartridge purification procedure is reported. In contrast to HPLC purification, the solid phase extraction (SPE purification) procedure was easier to include in radiosynthesis under good manufacturing practice (GMP) conditions. No modification of a commercial module was needed. The [^11^C]-(+)-α-DTBZ cartridge purification is easily transferable to other commercial synthesizers such as GE TracerLab FXc.

## References

[1] Zhang G., Dwoskin L.P., Crooks P.A. (2006). Vesicular Monoamine Transporter 2: Role as a Novel Target for Drug Development. AAPS J..

[2] Bohnen N.I., Albin R.L., Koeppe R.A., Wernette K.A., Kilbourn M.R., Minoshima S., Frey K.A. (2006). Positron emission tomography of monoaminergic vesicular binding in aging and Parkinson disease. J. Cerebr. Blood Flow Metab..

[3] Kilbourn M.R., Frey K.A., Borght T.V., Sherman P.S. (1996). Effects of dopaminergic drug treatments on in vivo radioligand binding to brain monoamine transporters. Nucl. Med. Biol..

[4] Kemmerer E.S., Jdesmond T., Albin R.L., Kilbourn M.R., Frey K.A. (2003). Treatment effects on nigrostriatal projection integrity in partial 6-OHDA lesions: Comparison of L-DOPA and pramipexole. Exp. Neurol..

[5] Anlauf M., Eissele R., Schäfer M.K., Eiden L.E., Arnold R., Pauser U., Klöppel G., Weihe E. (2003). Expression of the Two Isoforms of the Vesicular Monoamine Transporter (VMAT1 and VMAT2) in the Endocrine Pancreas and Pancreatic Endocrine Tumors. J. Histochem. Cytochem..

[6] Harris P.E., Ferrara C., Barba P., Polito T., Freeby M., Maffei A. (2008). VMAT2 gene expression and function as it applies to imaging β-cell mass. J. Mol. Med..

[7] Lang A.E., Koller W.C., Paulson G. (1995). Clinical rating scales and videotape analysis. Therapy of Movement Disorders.

[8] Tong J., Wilson A.A., Boileau I., Houle S., Kish S.J. (2008). Dopamine Modulating Drugs InfluenceStriatal (+)-[^11^C]DTBZ Binding in Rats:VMAT2 Binding is Sensitive to Changes in Vesicular Dopamine Concentration. Synapse.

[9] Jewett D.M., Kilbourn M.R., Clee L. (1997). A simple synthesis of ^11^C-dihydrotetrabenazine (DTBZ). Nucl. Med. Biol..

[10] Quincoces G., Collantes M., Catalan R., Ecay M., Prieto E., Martino E., Blesa F.J., Álvarez-erviti L., Areses P., Arbizu J. (2008). Quick and simple synthesis of ^11^C-(+)-a-dihydrotetrabenazine to be used as a PET radioligand of vesicular monoamine transporters. Rev. Esp. Med. Nucl..

[11] Zhang J.M., Tian J.H., Wang W.S., Liu B.L. (2004). An automated synthesis of ^11^C-methyl iodide with single vessel. Chin. J. Nucl. Med..

[12] Zhang J.M., Tian J.H., Wang W.S., Liu B.L. (2006). On-line Production of ^11^C-CH_3_-Triflae. Isotopes.

